# Paradoxical sleep deprivation impairs mouse survival after infection with malaria parasites

**DOI:** 10.1186/s12936-015-0690-7

**Published:** 2015-04-28

**Authors:** Lisandro Lungato, Marcos L Gazarini, Edgar J Paredes-Gamero, Sergio Tufik, Vânia D’Almeida

**Affiliations:** Department of Psychobiology, Universidade Federal de São Paulo, Rua Napoleão de Barros, 925, 3rd floor, São Paulo, SP 04024-002 Brazil; Department of Biosciences, Universidade Federal de São Paulo, Santos, SP Brazil; Department de Biochemistry, Universidade Federal de São Paulo, São Paulo, SP Brazil

**Keywords:** *Plasmodium chabaudi*, Sleep deprivation, Rebound, Parasitic infection, Experimental animals, Blood

## Abstract

**Background:**

Parasitic diseases like malaria are a major public health problem in many countries and disrupted sleep patterns are an increasingly common part of modern life. The aim of this study was to assess the effects of paradoxical sleep deprivation (PSD) and sleep rebound (RB) on malarial parasite infection in mice.

**Methods:**

After PSD, one group was immediately infected with parasites (PSD). The two other PSD rebound groups were allowed to sleep normally for either 24 h (24 h RB) or 48 h (48 h RB). After the recovery periods, mice were inoculated with parasites.

**Results:**

The PSD group was the most affected by parasites presenting the higher death rate (0.02), higher number of infected cells (p < 0.01), and decrease in body weight (p < 0.04) compared to control and 48 h RB groups. The 24 h RB group was also different from control group in survival (p < 0.03), number of infected cells (p < 0.05) and body weight (p < 0.04). After 48 hours of sleep rebound animals were allowed to restore their response to parasitic infection similar to normal sleep animals.

**Conclusions:**

These results suggest that PSD is damaging to the immune system and leads to an increased infection severity of malaria parasites; only 48 hours of recovery sleep was sufficient to return the mice infection response to baseline values.

**Electronic supplementary material:**

The online version of this article (doi:10.1186/s12936-015-0690-7) contains supplementary material, which is available to authorized users.

## Background

In a recent review about sleep duration and mortality Grandner *et al.* [1] pointed that in general, a U-shaped fashion association occurred between sleep duration and mortality, such that the lowest risk is found in individuals who report sleep durations of 7–8 hours. Individuals who got less than five or those who got ten or more hours of sleep per night had higher than average death rates [1]. The findings presented in this review have been reinforced by extensive scientific literature showing that the sleep is essential for the integrity and physiological maintenance of living beings, and sleep deprivation has been shown to have harmful effects in both humans and experimental models. Several studies have shown a close correlation between sleep and the immune system [2,3]. Sleep deprivation is responsible for the suppression of responses against antigens [4-6], a reduction in total leukocytes and lymphocytes in rats undergoing sleep restriction for 21 days [7] and reduced NK cell activity in partially sleep-deprived humans [8].

Guariniello *et al.* [9] observed a reduction in the total cell number within bone marrow and a decrease in granulocytes and monocytes that was concomitant with lymphopenia in the peripheral blood of sleep-deprived mice. However, other studies have shown an increase in circulating phagocytes accompanied by an increase in pro-inflammatory molecules, such as IL-1 and MHCII [10] as a result of sleep deprivation. There is a complex relationship between sleep, the central nervous system and immune system function, which involves neuropeptides, cytokines and microbial products [4] as well as Toll-like receptors (TLRs), endogenous ligands [11] and markers of activated macrophages, such as chitotriosidase [12].

Malaria is a parasitic disease that is common in tropical countries and is considered an important public health problem [13,14]. *Plasmodium chabaudi* is a murine parasite used for experimental malaria research. It is considered a genetically attenuated strain that is generated through many cycles within a mosquito vector, the intermediate host [15,16], which can quickly evolve into a more virulent form when in contact with the definitive host [17]. Studies have shown that there is an increase in the infectious process accompanied by the production of interferon gamma (IFNγ), which facilitates a response to TLR agonists, followed by an increase in serum IgG titers of cured mice [18,19]. Thus, the immune system provides malarial resistance in the infected animal through different mechanisms, which ensures a more efficient healing response [20].

Despite the large number of studies on the effects of sleep and the severity of viral and bacterial infections [2,4,21,22], to the best of recent knowledge, no studies have explored the relationship between sleep and protozoal infections. In this context, the present study adds evidence concerning the consequences of PSD and the effect of recovery sleep on the immune response during parasitaemia caused by *P. chabaudi* malaria parasites. Since sleep deprivation is a common event in modern human life and malaria is a public health problem in tropical countries these findings highlight the importance of good sleep in preventing the progression of parasitic diseases.

## Methods

### Animals

Forty male Swiss mice (3 months of age) from the colony maintained by the Department of Psychobiology - Universidade Federal de São Paulo (UNIFESP) were used in this study. Animals were maintained on a light–dark 12:12 cycle under controlled temperature conditions (20 ± 2°C) with free access to food and water. This study was carried out in strict accordance with the recommendations in the Guide for the National Committee for Researches Ethics (CONEP). The protocol was approved by the Committee on the Ethics of Animal Experiments of the Universidade Federal de São Paulo - UNIFESP (Permit Number: 0183/08). The animals used in this study were maintained and treated in accordance with the guidelines established by the Ethical and Practical Principles of the Use of Laboratory Animals [23] where, predicts the minimum of suffering for animals. The animals were separated into four groups: control (CT), paradoxical sleep-deprived (PSD) for 72 hours, 24-hour rebound group (24 h RB) and 48 hour rebound group (48 h RB) after sleep deprivation.

### Sleep deprivation

Three groups of 10 animals each were deprived of sleep for 72 h using the multiple platform technique, which was modified and adapted to mice [24]. They were placed on a small platform (3 cm diameter) surrounded by water in a container (41 cm × 34 cm × 16.5 cm), so that they were awakened by muscle atonia after touching the water. After PSD, one group was allowed to sleep normally for 24 h and another for 48 h, thus establishing the rebound groups. A group (n = 10) placed in the same environment, but in home cages was used as control and had normal sleep.

### Malaria parasites infection

After the experimental procedure of sleep deprivation and recovery periods, all groups, including the control, were inoculated with murine malaria-causing parasites (*P. chabaudi*). Mice were inoculated intraperitoneally with 1 x 10^6^ infected red blood cells (iRBC) and about 20% parasitaemia at trophozoite stage [25]. Blood was collected from the tail after 8 days following parasite inoculation [15] and placed onto microscope slides using the smear technique. Parasitaemia was determined by Giemsa staining and was examined by light microscopy in oil immersion magnified 1,000 times. The survival of the animals was followed for 20 days after infection by the parasites. During this period animals were observed twice a day by the researchers in order to verify their health conditions and avoid suffering. Until day 8 all of them did not present symptoms. After that, some animals showed less locomotor activity and tremor probably due the infection and some of them died. The 24 h RB group had more than one episode of symptoms followed by death. After trial period, the surviving animals were euthanized by decapitation.

### Counts of parasites

The number of infected cells was counted from the 8^th^ through to the 13^th^ day of survival after inoculation. A minimum of three different fields of view of the microscope objective and a total of approximately 1,000 cells were evaluated. The total amount of parasitized and non-parasitized cells and, subsequently, the percentage of infected cells for each group on every day of their collection were determined.

### Measures of body weight

To verify body weight changes during sleep deprivation, fourteen mice were distributed in two groups: control and PSD for 72 h followed by 24 hours of normal sleep. These animals had their body weights recorded every day during the sleep deprivation and recovery procedures.

### Statistical analysis

For cumulative survival experiments, statistical significance between the CT and experimental groups were analysed using the Kaplan Meier curve and Log-rank (Mantel Cox) test. The statistical analysis of counting parasitized cells was performed by one-way ANOVA, followed by the Newman Keuls post hoc test. The measures of the body weight of the mice were analysed by Student’s paired t – test. Also regression was performed to evaluate the correlation (Pearson coefficient) between the number of parasitized cells and the survival of animals. Calculations were performed using PrismTM version 4.03 for Windows (GraphPad, Software Inc. 7825 Fay Avenue, Suite 230 La Jolla. CA, USA).

## Results

### Survival curves of mice infected with *Plasmodium chabaudi*

**Figure 1:**
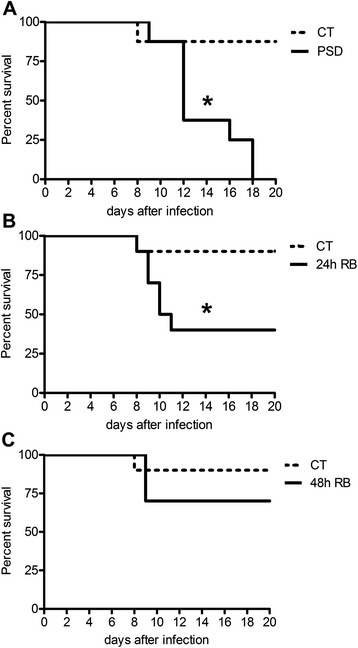
Survival curve of mice infected with a murine malaria protozoa (*Plasmodium chabaudi*) for 20 days after infection. **A**: Comparison of infected mouse survival between the CT and PSD groups. p < 0.015, Log-rank (Mantel Cox) test (n = 10). **B**: Comparison of infected mouse survival between the CT and 24 h RB groups. p < 0.03, Log-rank (Mantel Cox) test (n = 10). **C**: Comparison of infected mouse survival between the CT and 48 h RB groups. p > 0.38, Log-rank (Mantel Cox) test (n = 10). The results are expressed as the mean ± S.E.M.

The survival of mice was monitored for 20 days. The PSD group did not resist parasite proliferation during the infectious process (Figure 1A). By the 18^th^ day following inoculation, all mice from the PSD group had died, but the CT group remained with nine animals (only one died). When comparing the 24 h RB group to the CT group, we also noticed a significant reduction in survival, though 40% of the animals remained alive (Figure 1B). Interestingly, the 48 h RB group demonstrated restored rates of survival compared with the CT group (Figure 1C), as analyzed by the cumulative survival curve calculated using the Kaplan-Meier method.

### Percentage of cells parasitized during the infectious process

**Figure 2:**
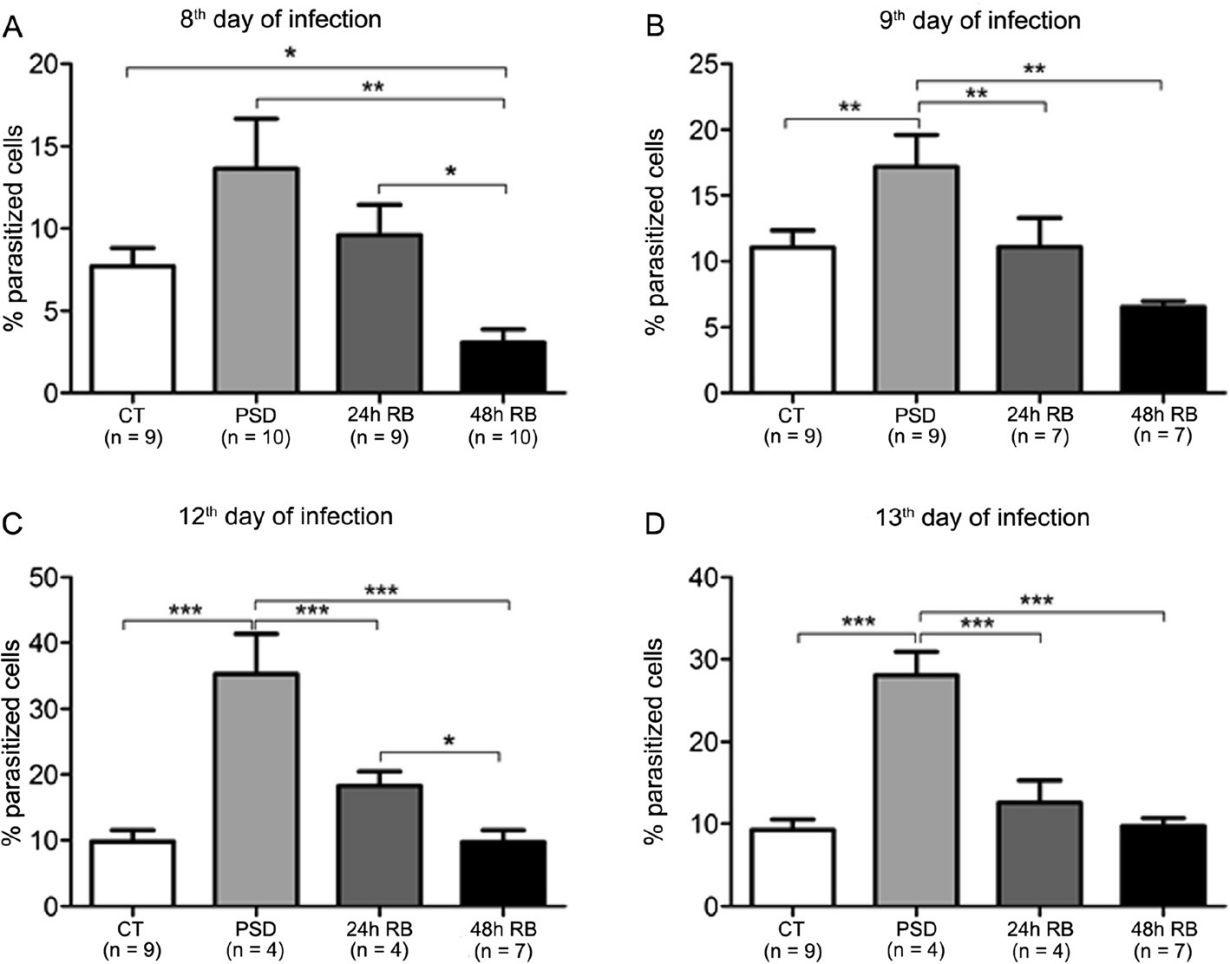
Parasitized erythrocytes (% of total cells) of mice infected with *P chabaudi.* **A**: 8^th^ day after infection. F_(3.35)_ = 6.04, p < 0.0004, one-way ANOVA test followed by post hoc Newman Keuls (* p < 0.05; **p < 0.01). **B**: 9^th^ day after infection. F_(3.29)_ = 8.672, p < 0.02, one-way ANOVA test followed by post hoc Newman Keuls (**p < 0.01; ***p < 0.001). **C**: 12^th^ day after infection. F_(3.17)_ = 18.95, p < 0.0001, one-way ANOVA test followed by post hoc Newman Keuls (* p < 0.05; ***p < 0.001). **D**: 13^th^ day after infection. F_(3.13)_ = 14.24, p < 0.0001, one-way ANOVA test followed by post hoc Newman Keuls (***p < 0.001). The results are expressed as the mean ± S.E.M.

Because the parasite takes an average of 8 days to establish acute proliferation in the bloodstream of mice, blood collection was started on the 8^th^ day of inoculation. On this day, the infectious process showed no difference in survival percentage between the PSD and the CT groups. However, all groups presented significantly higher levels of infected cells compared with the 48 h RB group, including the CT group (Figure 2A). On the 9^th^ day of the infectious process, a significant difference in the percentage of parasitized cells in the PSD group compared with all other groups (CT, 24 and 48 h RB; Figure 2B) was observed. Moreover, on day 12, an increase in the number of parasitized cells in the PSD group compared with all other groups also occurred. This increase in the number of parasitized cells occurred in association with a large decrease in survival probability. Furthermore, a reduction in the number of parasitized cells in the 48 h RB group compared with the 24 h RB and CT groups (Figure 2C) has been observed. Regarding the group PSD results, on day 13, a continued increase in the number of parasitized cells was observed compared with that of all other groups (Figure 2D). The correlation between the number of parasitized cells and the animal survival (days alive after infection) of each group was evaluated and a significant negative correlation was observed considering the four groups as a whole (r = −0,59; Table 1) and, also, when each group was analyzed separately.

**Table 1:** **Correlation between animals survival time versus number of infected cells**

**Pearson’s coefficient (parasitized cells number on 8th day vs survival time of the mice)**		
All groups	r = -0.59	p = 0.00006
Control	r = -0.73	p = 0.019
PSD	r = -0.65	p = 0.04
24 h RB	r = -0.80	p = 0.005
48 h RB	r = -0.70	p = 0.023

There are negative correlations (Pearson coefficient) between all groups (CT: r = −0.73, PSD 24 h: p = −0.65, RB 24 h: r = −0.80 and, RB 48 h: r = −0.70, respectively) as well as considering all animals independent the experimental group (r = − 0.59, p = 0.00006).

### Body weight of animals

During the monitoring of the animals body weight the PSD group presented reduced values and remained low even after recovery of 24 hours of sleep recovery compared to the control group (Additional file 1: Figure S1).

## Discussion

Pioneering experimental work carried out in the XIX century using dogs showed that total sleep deprivation was fatal for these animals. In these studies, brain histological sections presented severe lesions such as degeneration of neurons in the spinal ganglia and frontal cortex in the Purkinje cells of the cerebellum [26]. Sleep deprivation is an event that leads to dysfunction, stress and immune system suppression [6-9], and it is concomitant to the loss of the physiological, cellular and molecular integrity [27-30]. The suppression of the immune system associated with excessive inflammation was described by Langhorne *et al.* [31] as a key factor in the development of severe malaria. Also it was shown that histological analysis of the liver of mice infected with malaria parasites on day 7 showed extensive inflammation in sinusoidal vessels and damage to hepatocytes with nuclear fragmentation and vacuolation [32]. These mechanisms could explain why in our study all animals in the PSD group (100%, Figure 1A) died after 18 days of infection, because sleep is a crucial component in an efficient immune response [2,27]. The mortality rates remained high, even with 24 h RB, before the inoculation of the parasite into mice (60% mortality, Figure 1B). The 48 h RB group was able to control the parasitic infection and had lower mortality (30%, Figure 1C), which, in turn, corroborated the role of sleep in contributing to the effectiveness of the immune system [28,30] and in the restoration of the response against pathogens.

Everson [28] observed extravascular migration of neutrophils in liver and lung tissue concomitant with cellular stress, tissue injury and induced infection in sleep-deprived rats. During the peak of parasitaemia, Medeiros *et al.* [32] noticed an excessive intravascular haemolysis that is responsible for increased oxidative stress and the reduced expression of MHCII in lymphoid dendritic cells. Interestingly, for the count of the infected cells, there was an increase of approximately 85% in the PSD group compared with the CT group (Figure 2A) in the present study. This result may suggest that the immune system is not able to control the parasite infectivity under sleep loss conditions. Previous work showed that circulating levels of TNF and IL-2 are altered in many diseases in which sleep is insufficient, including chronic inflammation, excessive daytime drowsiness and HIV and influenza virus infections [4,33,34]. The TNF and IL-2 cytokines are pro-inflammatory activators in cases of infections [34] and it is possible that their levels are elevated during parasitaemia, which, in turn, could be an explanation for our results. The parasitic infection capacity remained high at day 9 (Figure 2B), day 12 and day 13, where the parasitic load in PSD animals was even greater, with increases of 120% and 149%, respectively for day 12 and 13 (Figure 2C and D). These data indicate the weakening of the immune system caused by sleep loss, which supports the survival results found until the 18^th^ day of infection (Figure 1A). Although there are no reports about the immunological mechanisms of parasitaemia and sleep deprivation, many studies show an immunodeficiency against infections during sleep loss [2,21,27,30], which possibly explains greater vulnerability to infections from parasites as we found in our this study. It was demonstrated in a previous work published by the occurrence of mitochondrial and lysosomal dysfunction with changes in ion homeostasis [29] and oxidative stress in splenic cells of PSD mice [35]. These results corroborate the present findings and show that the impairment of tissue and cellular physiology caused by a loss of sleep concomitant to the damage caused by parasitic infection is strong evidence for immune suppression being responsible for the eventual death of these animals.

Although most animals in the 24 h RB group also succumbed during infection, no difference was observed in parasitic burden compared with the CT group. Only on day 12 did the parasitic rate increase 32%. Moreover, there was a negative correlation between the survival rates *versus* the number of parasitized cells over time, which may indicate the sleep loss as a contributor to the animal death (Table 1) since PSD group presented a higher percentage of parasitic cells from the 9^th^ day of infection (Figure 2). These findings raise two hypotheses: I – mice that were severely affected by sleep loss died and therefore did not contribute as individuals to the count of the infected cells; and II – the recovery of sleep for 24 h restored the efficiency of the immune system to some extent, but this came at the cost of a high energy expenditure, as evidenced by their weight loss, which put this group at a disadvantage in terms of survivability when compared to the CT group (Additional file 1: Figure S1). Corroborating this second hypothesis, previous studies have shown that sleep deprivation causes symptoms of secondary innutrition and mortality in experimental models [10,28,36]. Martins *et al.* [37] observed a reduction in body weight in rats subjected to 24 h of sleep RB after PSD, even those receiving a high fat diet. It has been previous demonstrated that PSD and 24 h RB were associated with weight loss in animals, which could be explained by the enhanced energy expenditure suffered during the experimental protocol (Additional file 1: Figure S1). These effects of sleep loss, which results in energy deficits, could be an explanation for the reduced capacity of animals to handle parasitic infection.

The evidence cited above and the variety of articles reporting immunosuppression caused by poor sleep may provide evidence that 24 hours of recovery sleep is not enough to restore the homeostatic integrity of the immune system. These results support the present findings, which showed that even after 24 hours of recovery sleep the animals did not respond efficiently against parasite infection.

The efficiency of the immune system after 48 h of recovery sleep is remarkable. In the 48 h RB group, only three deaths occurred until day 9, which is known as the day after the peak of parasitic infection according to the findings of Pollitt *et al.* [15] and Spence *et al.* [16]. It is probable that these were animals that failed to restore physiological and immunological integrity for a sufficient time to effectively respond to a pathogenic infection. However, the immune efficiency of 48 h RB mice seems to be higher than the CT group because it was observed a reduction in the number of infected cells (65% on day 8 and 33% on day 9; Figure 2A and B). This observation suggests that the increase in sleep duration and changes in sleep architecture, which is a characteristic of the recovery period [38-40], could produce an effect in improve the immune response.

The reduction of the parasitic load was reduced on days 12 (57%) and 13 (60%) compared with the CT group (Figure 2C and D). This is reinforced by the negative correlation between survival versus number of parasitized cells over time in these animals (Table 1). These data suggest the extent to which sleep is important for the effectiveness of the immune system. Accordingly, Hirotsu *et al.* [30] observed that mice undergoing psoriasis and sleep deprivation were able to respond only after 48 hours of recovery sleep. The findings obtained in the present study for the 48 h RB group were also corroborated by Everson and colleagues [28], who observed an increase in myeloperoxidase activity in the liver and lungs of rats that were sleep-deprived and returned to normal activity levels after 48 h of sleep rebound. These results reinforce the importance of sleep and its recovery in the efficiency and integrity of the immune system.

## Conclusions

Several studies have shown that sleep is essential to the efficiency of infectious disease recovery but in modern life people are increasingly being sleep deprived due to their needs and lifestyle. Parasitic diseases are still a public health problem of high frequency in a number of countries in tropical regions. For this reason, is important to investigate parameters of quality of life, such as good nights of sleep and the effects of sleep loss in people at risk.

The present data suggest that PSD caused an increase in the infection by malaria parasites, decreasing the rate of mice survival. PSD, even with recovery for 24 h, was possibly responsible for the death of animals infected with malaria parasites, increasing counts of parasitized cells in the blood and body weight loss. In addition, only after 48 h of sleep recovery the animals were able to resist the pathogen infection. These data highlight the need to increase awareness about the role of sleep in recovery from infectious processes.

## Additional file

Additional file 1: Figure S1. Body weight in animals during experimental protocol. There are differences in weight of the animals after 72 hours of PSD and 24 hours of rebound after PSD (*p <0.04) compared to the control group. The results are expressed as the mean ± S.E.M.
