# Supplementary figures and images for: Paradoxical sleep deprivation impairs mouse survival after infection with malaria parasites
Source: Malar J. 2015 Apr 28;14:183. doi: 10.1186/s12936-015-0690-7 (PMC4416287; doi:10.1186/s12936-015-0690-7)

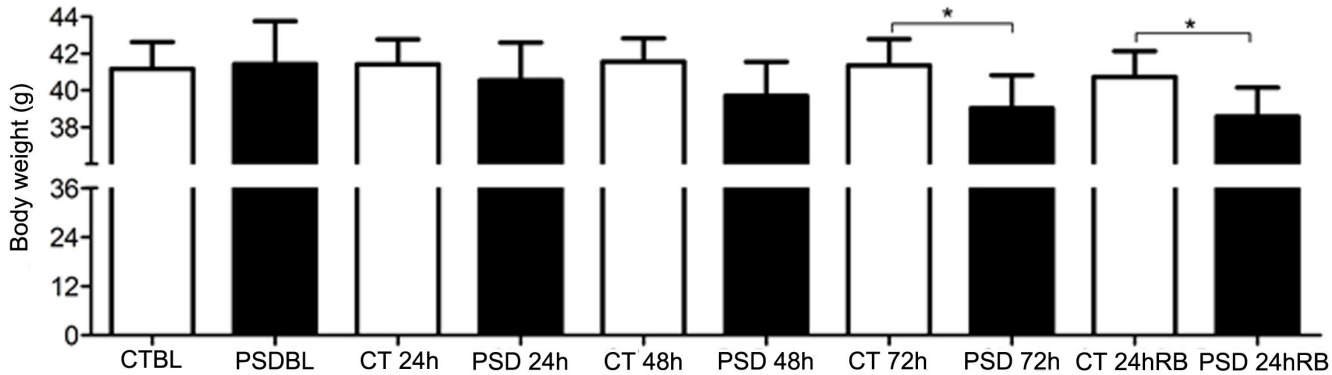

Supplement: Additional file 1: Figure S1. — Body weight in animals during experimental protocol. There are differences in weight of the animals after 72 hours of PSD and 24 hours of rebound after PSD (*p <0.04) compared to the control group. The results are expressed as the mean ± S.E.M. [file 12936_2015_690_MOESM1_ESM.pdf]
